# Supporting physical activity engagement in people with Huntington’s disease (ENGAGE-HD): study protocol for a randomized controlled feasibility trial

**DOI:** 10.1186/1745-6215-15-487

**Published:** 2014-12-12

**Authors:** Monica Busse, Lori Quinn, Helen Dawes, Carys Jones, Mark Kelson, Vincent Poile, Rob Trubey, Julia Townson, Rhiannon Tudor Edwards, Anne Rosser, Kerenza Hood

**Affiliations:** School of Healthcare Sciences, Ty Dewi Sant, Heath Park Campus, Cardiff University, Cardiff, CF14 4XN UK; Department of Sport and Health Sciences, Oxford Brookes University, Headington Road, Oxford, OX3 0BP UK; Centre for Health Economics and Medicines Evaluation, Ardudwy Building, Bangor University, Bangor, LL57 2PZ UK; South East Wales Trials Unit, Cardiff University, Neuadd Meirionydd, Heath Park Campus, Cardiff, CF14 4YS UK; Schools of Medicine and Biosciences, Cardiff University, Park Place, Cardiff, CF10 3BB UK

**Keywords:** Huntington’s disease, Physical activity, Randomized controlled feasibility trial

## Abstract

**Background:**

Huntington’s disease (HD) is a complex, single-gene inherited neurodegenerative condition resulting in symptoms that occur across a wide range of neurological domains, including cognitive, behavioral and motor. The benefits of regular physical activity for people with HD are widely recognized. However, a number of factors can prohibit sustained exercise and activity. The purpose of this trial is to explore the feasibility, acceptability and effectiveness of a physical activity intervention program targeted for people with early- to mid-stage HD.

**Methods/Design:**

The proposed trial is a single blind, multisite, exploratory, randomized controlled feasibility trial of a physical activity intervention. A total of 62 participants with genetically confirmed HD will be recruited. Each participant will be involved in the trial for 26 weeks. Participants will be randomized immediately following the baseline assessment into either a physical activity intervention or a social contact control intervention. The physical activity intervention is framed around self-determination theory placed within a broader behaviour change wheel framework. An HD-specific workbook and individual goal setting will be utilized over six 1:1 sessions, with interim telephone calls. All participants will be reassessed at 16 weeks following the baseline assessment, and then again at a final follow-up assessment 26 weeks later. At the end of the study, all participants will be offered a brief version of the alternative intervention, with one home visit and one follow-up telephone call.

**Discussion:**

Engaging and supporting people with HD in a regular physical activity program raises a number of challenges. The physical activity intervention and the comparator social interaction intervention have been developed following consultation with people with HD and their families. Each are individually tailored and determined on individual needs and goals. The results from this trial will provide guidance for the development of definitive trials.

**Trial registration:**

The trial was registered with ISRCTN ( http://www.isrctn.com/ISRCTN65378754) on 13 March 2014.

## Background

Huntington’s disease (HD) is a complex, single-gene inherited condition that produces gradually progressing symptoms, usually from mid-life over a 15 to 20-year-period. Symptoms occur across a wide range of neurological domains, including cognitive, behavioral and motor [1]. The slowly progressive nature of HD and the current lack of successful disease-modifying interventions means that relatively young people with the condition are often dependent on assistance for activities of daily living. Impact on quality of life and caregiver burden is often great due to the physical and emotional dependency that develops with greater disease severity [2–6].

With neurodegenerative diseases, it is now widely recognized that regular and sustained physical activity has the potential to benefit cardiovascular health [7] and multiple aspects of physical functioning, including postural control, gait and health-related quality of life [8–10]. Research has shown a number of factors which impact on physical activity participation, including fluctuations in physical health, transportation issues, other time conflicts, social stigma, external demands and lack of motivation [11–13]. In addition, these issues are often further compounded by disease-specific barriers in the presence of neurodegenerative disease such as HD. Provision of appropriate support and strategies that are modified to the specific nature of the disease are crucial, as people with HD may struggle to participate in regular conventional physical activity or exercise programs [14]. In the case of HD, challenges are specific to the triad of motor, cognitive and behavioral symptoms that are notably complicated by apathy alongside difficulties in planning and executive function.

Here we present the protocol for a single blind, multisite, exploratory, randomized controlled feasibility trial of a physical activity intervention. The aim of the trial is to explore the feasibility and acceptability of this intervention for people with early- to mid-stage HD. A range of outcomes will be explored to assess benefit. As it is possible that many of the beneficial effects reported in physical activity interventions are as a direct result of social activity, the study incorporates a social comparator arm, involving a structured social intervention, which is equally matched for contact time.

## Methods/Design

### Study design

**Figure 1:**
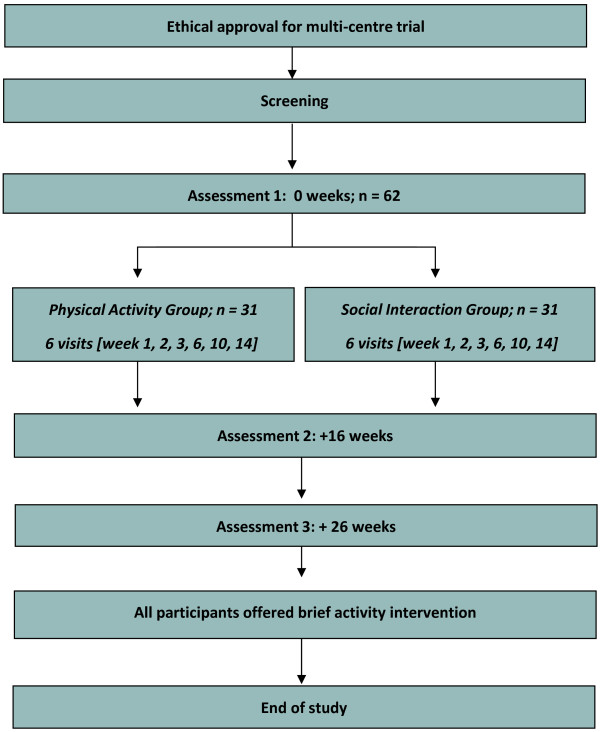
**Study schema.**

The proposed trial is a single blind, multisite, exploratory, randomized controlled feasibility trial of a physical activity intervention. The comparator is a social interaction intervention. The trial schema is illustrated in Figure 1.

### Site and participant selection

The trial will be carried out in eight HD specialist clinics, most of which are Registry/Enroll-HD (NHS Research Ethics Committee approval numbers: 04/WSE05/89; 13/WA/0192) sites for the European Huntington’s Disease Network (EHDN). Many patients attending the HD clinics are already enrolled in the Registry/Enroll-HD study. The progression in symptoms of these patients has been followed by clinicians and researchers for a number of years. The Registry/Enroll-HD study is a full clinical dataset, including full medical history and medication history. One of the optional components within the Registry project is the giving of permission by participants to be contacted about other additional and affiliated HD research projects. In consenting to be enrolled in the Registry/Enroll-HD study, participants also give their permission for their coded data to be accessed by researchers conducting other HD-related research.

### Inclusion and exclusion criteria

Participants will only be eligible for the trial if they meet the inclusion and exclusion criteria in Table 1.

**Table 1:** **Participant inclusion and exclusion criteria**

Inclusion criteria	Exclusion criteria
1. Diagnosis of manifest Huntington’s disease, confirmed by genetic testing	1. Any physical or psychiatric condition that would prohibit the participant from completing the intervention or the full battery of assessments
2. Self-reported or physician-reported difficulties with walking and/or balance (but still able to walk with minimal assistance)	
	2. Unable to understand or communicate in spoken English
3. Over 18-years-old	3. Currently involved in any interventional trial or within four weeks of completing any interventional trial
4. Stable medication regime for four weeks prior to initiation of trial, and anticipated to be able to maintain a stable regime for the course of trial	

### Recruitment

We aim to recruit 62 participants. At each site, eligible patients receiving routine HD clinical care or attending for a Registry/Enroll-HD research assessment will be given information about the trial and an invitation letter describing the trial by the clinician responsible for their care. All interested potential participants will be given a minimum of 24 hours to read the material and discuss with their families and carers before any follow-up telephone call, as per the Registry/Enroll-HD study protocol. They will have the opportunity to ask any questions they have about the trial and discuss their potential involvement in the trial.

An invitation letter and information sheet will be sent to those Registry/Enroll-HD patients who may be eligible, but not due to visit the clinic imminently (these individuals will be identified from the Registry database with the assistance of Registry/Enroll-HD investigators at the site). Telephone follow-up as per the Registry/Enroll-HD study protocol will then be conducted. The study is eligible for the National Institute for Health Research Clinical Research Network Portfolio (NIHR CRN). It is possible that potential participants could see the trial described on this organization’s website and may express an interest in being involved. If any person does self-refer to the trial team, they will be given an information sheet, have the opportunity to ask any questions they have about the trial and discuss their potential involvement in the trial. If they are eligible and not enrolled in the Registry study, potential participants would be required to be fully reviewed by the site Principal Investigator (PI) to be assured that the inclusion and exclusion criteria are met. This includes the availability of genetic confirmation and a recent HD specialist assessment confirming the status of the disease.

### Consent

Informed consent will be obtained from all participants, including consent to be randomized to either the physical activity or social interaction groups. Withdrawal of consent will have no detrimental impact on current and future treatment. If a participant were to lose their capacity to consent during the trial, the subject will be withdrawn and no further procedures will be carried out on the participant. No new personal data will be collected. Data that has already been collected in relation to the participant may be retained and used for the purposes for which consent has already been given, provided they are effectively anonymized and no longer identifiable to the research team.

### Randomization

Once informed consent is obtained and the first baseline assessment completed, randomization will be performed by site staff using the online database. The randomization ratio to the physical or social intervention will be 1:1. A minimization technique will be used to achieve, as far as possible, balance between groups based on data obtained at the baseline assessment. Variables used for minimization will be: site of recruitment; age (less than 50-years-old or 50-years-old or greater); gender; Unified Huntington’s Disease Rating Scale (UHDRS) total motor score (less than 45 or 45 or greater).

### Blinding

This is a single blind trial. All data collection will be conducted by a team of blinded assessors specifically trained in the methodology utilized for the collection of physical activity and functional assessments. The participants will be requested by the liaison person to not disclose their allocation to the assessors. Records of incidents where blinding is broken will be kept.

### Interventions

#### Physical activity intervention

**Figure 2:**
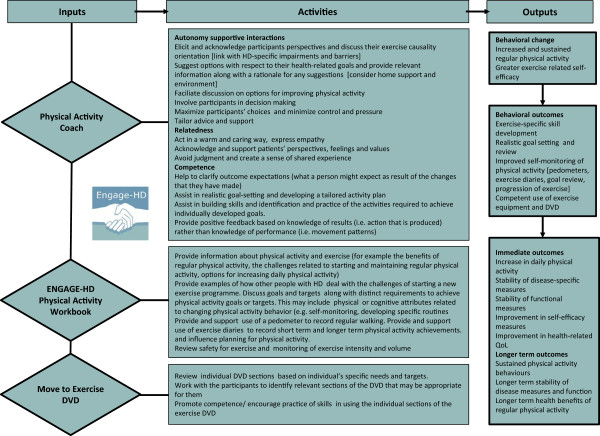
**Logic model for the ENGAGE-HD physical activity intervention.** HD, Huntington’s Disease; QoL, Quality of Life.

The ENGAGE*-*HD physical activity intervention consists of three main elements, namely the physical activity coach, a purpose developed exercise DVD (*Move to Exercise*) (Cardiff University, Cardiff, United Kingdom) [10, 15] and a purpose-developed physical activity workbook (Figure 2) (Cardiff University, Cardiff, United Kingdom). The intervention will specifically focus on developing an individualized lifestyle approach to enhancing physical activity, where interpersonal interactions of the physical activity coach are underpinned by the concepts of self-determination theory (SDT). SDT conceptualizes motivation for physical activity along a continuum, ranging from activity that is extrinsically motivated and regulated (in order to gain rewards or to satisfy an external demand), to activity which is intrinsically motivated (autonomous, self-determined behaviour). Conditions that lead to enhanced and more self-determined motivation include a supportive social environment that accommodates individual needs, choices and perspectives, and encourages competence [16, 17]. The function of the additional intervention components, namely a physical activity workbook and exercise DVD, are to facilitate education, enablement, modelling and goal setting. They are therefore more appropriately placed within a broader behaviour change wheel (BCW) framework [18], which focuses on the interactions between capability (physical and psychological), opportunity (physical and social) and motivation (intrinsic and extrinsic) that produce the observed behaviour.The logic model developed for this study is provided in Figure 2. This figure illustrates the key elements (inputs), activities and outcomes of the physical activity intervention for this study.

Participants enrolled in the ENGAGE-HD physical activity intervention will receive six home visits and interim telephone calls over a course of 14 weeks, during which time they will be supported in developing an individualized, lifestyle approach to enhancing physical activity. These physical activity sessions will be delivered in each participant’s home by activity coaches, who will have been trained to deliver the specific intervention protocol. All activity coaches will either be health professionals who are able to demonstrate the relevant competencies for supporting exercise-related activities as set out within the NHS Knowledge and Skills Framework, or at least Level 3 Register of Exercise Professionals (REPS) registered exercise professionals who can provide clear evidence of meeting these specific competencies. The intervention delivery will be overseen by the lead intervention coordinator for the study, who is a registered physiotherapist. Each coach will undergo one-to-one training by the lead intervention coordination prior to the start of the trial at each site. In addition, the coach and intervention coordinator will dialogue on a regular basis, including at least one telephone call after a participant’s first visit, in order to review and discuss goals and the activity plan. Furthermore, one of the six sessions for each participant in the physical activity intervention will be audio recorded and reviewed by the lead therapist for the monitoring of intervention fidelity.

During the first home visit, the coach will introduce the participant to the ENGAGE*-*HD physical activity intervention, the workbook and the exercise diaries, which participants will be asked to complete each week. The initial interactions will consider benefits of physical activity and each participant’s individual exercise history, as well as setting specific physical activity goals. Further discussion topics on physical activity will include implementing a daily activity plan, monitoring exercise intensity, dealing with safety, weather, equipment and typical barriers (such as time, boredom, lack of equipment, lack of specific knowledge and support). In the remaining five home sessions, the coach will continue to support discussions related to the activities in the workbook, and supervise the participant performing components of the *Move to Exercise* DVD exercise program or other physical activities. Coaches will also review exercise diaries completed during the previous week(s). Supportive telephone calls will be conducted three times over the 16-week-period. These calls will serve to provide encouragement and advice with respect to the promotion of regular physical activity. During the calls, the researcher will also ask about any falls, health or medication changes and confirm the date and time of the next visit.

#### Social interaction comparator intervention

Participants assigned to the social interaction group will receive six visits over the same time period as the physical activity group. The sessions will also be matched for time (approximately 45 minutes). The aim of these sessions will be to provide conversational interaction. Each coach for the social interaction group will undergo one-to-one training with the lead intervention coordinator prior to the start of the trial at each site, and the intervention coordinator will be available for consultation throughout the trial. Each coach will meet with the participant in their home and will engage with the participant in a talking and communication interaction. Conversation cards (with images and text) representing a wide range of topics will be used by the researcher to help direct conversation toward topics of potential interest to the participants during each visit. In the first session, a ‘getting to know you’ conversation will take place. Further discussions may then focus on a range of topics including travel, media, food, music and art, entertainment, shopping, animals, science, technology, friends and socializing. At each visit, the coach will complete a health and falls review with the participant where they will ask about (and record any details of) any falls, health professional interaction or medication changes. Reminder telephone calls will be conducted three times over the 16-week-period. These calls will serve to match the contact time provided to the physical intervention group. During the calls, the researcher will also ask about any falls, health or medication changes and confirm the date and time of the next visit. At the final home visit, the participant will be assisted in completing a life-space assessment and a self-reported physical activity assessment. While both of these measures are included in the first and third outcome assessments, they will be gathered by intervention delivery staff at this time point in order to reduce the risk of un-blinding the assessment staff.

### Screening

A screening log will be kept at each site, where details of numbers of people who were approached about the trial, eligibility and whether consent to be contacted was given or declined will be recorded. A log of any individuals who declined at the initial consent stage will also be kept. We will ask the recruiting clinicians to keep a record of how many individuals have been approached in relation to the trial.

### Baseline measures

Demographic factors of age and gender and level of education will be assessed at baseline. Disease burden of pathology score, physical activity and social support (using the Social Support for Exercise Survey) [13, 19] will be documented. Participant height and weight, along with disease-specific measures of motor, cognition and function [20] will also be documented. Current medication at baseline and any medication changes at subsequent assessments will be recorded.

### Primary outcomes

The primary feasibility outcome will include an evaluation of eligibility, recruitment and retention rates (in line with CONSORT recommendations), as well as monitoring of completion of outcome measures and assessments. The safety of both interventions will be assessed via records review. Acceptability of the interventions will be assessed via structured questionnaires with research participants on completion of the intervention. Process evaluations will further assess whether the interventions were delivered as intended. This will be achieved via structured observation of intervention delivery, semi-structured interviews with intervention delivery staff and review of intervention records and participant diaries. These will be presented descriptively for each of the study groups.

### Secondary outcomes

A range of secondary measures will be explored in terms of short-term benefit. Details of which outcomes will be collected at the different time points are shown in Table 2 below.

**Table 2:** **Outcome measures**

Domain to be measured	Measure(s)
Measures of participation and health	
Individualized quality of life	Schedule for the evaluation of individual quality of life-direct weighting (SEIQoL-DW) [21]
Self-efficacy	Lorig self-efficacy scale [22]
Health service use	Client Services Receipt Inventory [23]
Health utility measures	EQ-5D-5 L [24]
	ICECAP-A [25]
Measures of activity	
Functional activity	Physical performance test (PPT) [26]
Usual pattern of mobility	Life space assessment [27]
Physical activity assessment	International Physical Activity Questionnaire (Short Form) [28]
Walking ability	Six-minute walk test [29]
	Timed Up and Go Test [30]
Self-reported falls	Frequency, circumstance and severity of any falls [31]
Measures of body function	
Disease-specific clinical measure of motor impairment	Unified Huntington Disease Rating Scale (UHDRS) modified motor scale [20]
Measure of cognitive impairment	Symbol Digit Modality Test (SDMT) [32]
	Verbal category fluency [33]
Behavioral outcomes	
Measures of autonomy/supportive interactions	PAS Healthcare Climate Questionnaire [34]

PAS, Perceived Autonomy Support; ICECAP, ICEpop CAPability measure for Adults.

The main assessment of short-term benefit will be provided by the Physical Performance Test (PPT) [26]. The test incorporates a series of timed tasks that are summed to give a score between 0 (severe problems) and 36 (minimal problems). Additional outcome measures include measures of physical activity and mobility in the community, self-efficacy, walking ability, health utility and quality of life. Self-reported physical activity will be measured using the International Physical Activity Questionnaire (IPAQ) [28]. Community engagement will be reflected by the Life Space Assessment [27]. The Lorig scale will provide a measure of self-efficacy [23]. Walking ability will assessed using the six-minute walk test [29] and the Timed Up and Go Test [30]. Participants will be asked to complete the EQ-5D generic health-related quality of life measure [24] and the "ICEpop CAPability measure for Adults" (ICECAP-A) generic health measure [25] via face-to face interview. An interview-administered individualized quality of life measure (SEIQol-DW) [21] will also be obtained at the final assessment. Self-reported frequency, circumstance and severity of any falls over the past four months will be recorded at assessments one and two, and over the past two months at assessment three. Health and social care services will be recorded using a Client Services Receipt Inventory (CSRI) [23] and participants will be asked to recall contacts (assisted by the main carer where possible). Where carer assistance is required, this will be documented. Behavioral outcomes will be assessed using the Perceived Autonomy Support (PAS) Healthcare Climate Questionnaire (short form) [34]. Standard clinical measures of disease severity, namely of motor [1] and cognitive impairment [32, 33], will also be obtained (see Table 2). Health and social care service use will be costed using national unit costs, so as to assess the incremental cost-effectiveness of the physical activity intervention compared to the social contact intervention. The ENGAGE-HD trial schedule of enrolment, interventions and assessments are summarized in Table 3.

**Table 3:** **Schedule of enrolment**, **interventions and assessments**

Study period														
	Enrolment		Allocation	Post-allocation									Follow-up	
Time point	-4 weeks	0	0	2 weeks	3 weeks	4 weeks	6 weeks	8 weeks	10 weeks	12 weeks	14 weeks	15 weeks	16 weeks	26 weeks
Enrolment														
Prescreening from research database	X													
Eligibility screen		X												
Informed consent		X												
Registration		X												
Par-Q safety screening		X												
Allocation			X											
Physical intervention:														
Physical intervention visits				X	X	X		X		X		X		
Audio recording of physical intervention visit						X								
Physical intervention group: review health and falls record					X	X		X		X		X		
Physical intervention group: review exercise diaries							X		X		X			
Telephone calls (physical intervention)							X		X		X			
Social intervention:														
Social intervention visits				X	X	X		X		X		X		
Social intervention group: review health and falls record					X	X		X		X		X		
Telephone calls (social intervention)							X		X		X			
Assessments:		X											X	X
Social support for exercise		X												
Physical performance test				X									X	X
Self-reported falls				X									X	X
UHDRS functional assessment				X									X	X
UHDRS modified motor assessment				X									X	X
Symbol digit modality test				X									X	X
Verbal category fluency				X									X	X
Six-minute walk test				X									X	X
Timed up and go test				X									X	X
IPAQ				X								X		X
EQ-5D				X									X	X
ICECAP-A				X									X	X
CSRI				X										X
Lorig self-efficacy scale				X									X	X
Life space assessment				X								X		X
PAS healthcare climate questionnaire													X	
SEIQoL-DW														X
End of study questionnaire														X

UHDRS, Unified Huntington’s Disease Rating Scale; IPAQ, International Physical Activity Questionnaire; CSRI, Client Services Receipt Inventory; SeiQOL-DW, Schedule for the Evaluation of Individual Quality of Life.

### Sample size

Based on preliminary studies, we can expect a mean standardized difference between those in the physical activity intervention group and those allocated to a control group (usual care) of 1.8 in the PPT. A more conservative effect size has been used in sample size calculations for this study to accommodate for: a) the inclusion of a social comparator arm in this study rather than no intervention (usual care) and b) the potential of a clustering effect in the single site pilot study. A total of 46 HD patients (23 per group) are sufficient to detect a more conservative standardized difference of 1.0 at the final measurement point, with a power of 90% and α of 0.05. We aim to recruit 62 subjects in total to allow for 25% loss to follow-up, based on retention rates from our previous studies. This will allow us to estimate any proportion to within 14.4 percentage points either side, using a 95% confidence interval.

### Fidelity

Intervention fidelity will be assessed in the following ways. On completion of each session, the coach will complete an intervention implementation checklist, reporting on the component activities of the intervention. At least one of the physical coaching sessions per participant will be audio or video recorded. The transcripts of these recordings will be used to analyze the quality of the intervention delivery. Fidelity of the coach interpersonal interactions will be measured by assessing the extent to which the coach’s interactions with the participant adhered to three elements of SDT, namely autonomy, competence and relatedness. Data from the PAS Healthcare Climate Questionnaire [34] completed by participants at assessment two will provide a measure of the individual coach’s perceived interaction style. Coach records and diaries will provide an assessment of use of the exercise DVD and the purpose-developed physical activity workbook.

### Data management

All assessment and intervention data will be entered by site staff to a study-specific online database, accessed via password-protected iPads (Apple Distribution International, Hollyhill Industrial Estate Hollyhill, Cork, Republic of Ireland) provided to each site. Sites will be provided with paper versions of the assessment and intervention forms to be used as a backup, but it is anticipated that most sites will enter data directly into the database.

Using the online database for data entry will reduce burden on site staff, and allows for immediate validation checks upon data entry, which will prompt staff to address any questions which have been missed or values which appear to be outside of an anticipated range. Site staff will have their own individual login details, which will ensure that they are only able to access forms and database functions which are appropriate for their staff role. Data monitoring will take place on 100% of completed forms shortly after submission, allowing for timely query generation and resolution and the minimization of missing forms or data.

In addition to being used for data entry, study iPads offer a number of benefits related to training, support and data monitoring. Following site initiation visits, site staff will be able to use the iPad to record videos of practice assessments, for instance, and send these to study members for feedback. The iPad can also be used by site staff to communicate with study members via Skype, allowing for ongoing support in delivering the intervention. Finally, the iPad can be used to audio record selected intervention sessions, which will be used for the aforementioned assessment of intervention fidelity.

### Safety monitoring

An adverse event (AE) is defined as any untoward medical occurrence in a trial participant. A serious adverse event (SAE) is any untoward and unexpected medical occurrence or effect that results in death; is life-threatening (refers to an event during which the participant was at risk of death at the time of the event); requires hospitalization, or prolongation of existing hospitalization; results in persistent or significant disability or incapacity, or is a congenital abnormality or birth defect. We do not anticipate any SAEs in this trial cohort, although there is a chance that some individuals with HD may require hospitalization in relation to the course of their disease. The most likely reasons for unrelated hospitalization in any person with HD would be due to respiratory problems and fractures due to falls. Falls are an expected AE as part of the clinical condition at this stage of the disease. Related SAEs or AEs are defined as any event that results from administration of any of the research procedures that are considered causal to the research process or intervention. While falls can occur in people with Huntington’s disease, we do not anticipate any increase in fall risk from the ENGAGE-HD physical activity intervention. Any falls will be recorded routinely by the research team and submitted for assessment of relatedness as part of the study protocol. The proposed activity intervention is low-to-moderate intensity and does not involve any heavy load-bearing exercise, heavy eccentric muscle activity or high intensity activities. However, some minor muscle soreness or musculoskeletal strain may occur in the few days following the initiation of physical activity. This would normally resolve spontaneously and would not require any specific interventions or additional medical care, but will be noted as a potential expected related AE if reported. All AEs, related adverse events (RAEs) and SAEs will be recorded using a standard template and reported in line with standard operating procedures.

### Exploratory statistical and health economics analysis

Summary statistics of demographics (age, gender, height and weight) and disease burden scores will be reported. Descriptive data will include an evaluation of eligibility, recruitment, retention rates and acceptability of, and adherence to the intervention, with 95% confidence intervals. Descriptive analysis of adherence rates according to baseline measures will be analyzed to inform the assessment of mediators of the intervention. The completion of outcome measures and assessments will also be reported. Graphical illustration will be used to check distributions of outcome data.

Successful adherence to the intervention will be defined as having completed visit one, two and three with their activity coach. Further aspects of adherence will be measured by the percentage of exercise and falls diaries completed by participants, and the percentage of participants who use the physical activity workbooks and exercise DVD. Descriptive analysis of goals set will be considered in relation to adherence. If retention rates are greater than the estimated 75%, we will consider this intervention to be feasible. If the proportion retained is less than this but greater than 65%, we will consider adjusting the intervention to increase this in future investigations. A retention rate lower than this would require substantial changes to the intervention and therefore would require further piloting.

Changes at the second assessment in all short-term secondary outcomes will be analyzed using analysis of covariance (ANCOVA) with the baseline score of that variable in addition to the balancing variables (age, gender and UHDRS motor score) as covariates. A logistic regression analysis will investigate whether the number of falls experienced differs between the treatment arms. This will be extended to joint modelling of both falls and physical functioning if there is a significant difference in the number of falls experienced. Data may be transformed to improve model fit, or different regression approaches used (negative binomial or Poisson regression). Descriptive analysis of data collected at the third assessment will be conducted with a view to assessing any sustained benefit of the intervention (if benefit is present at assessment two). All these analyses will be on an intention to treat (ITT) basis, although the primary analysis will use the complete case data set. In order to make ITT inferences possible, we will differentiate intervention discontinuation from trial withdrawal and, in the case of discontinuation, will encourage sites to continue to collect outcomes data wherever possible. Data collection will be performed on iPads and data completeness will be monitored at the point of collection, therefore we do not expect large amounts of missing data. Multiple imputation of covariates will be performed if the proportion of missingness exceeds 15%. A Complier-Average Causal Effect (CACE) will also be estimated using multilevel mixture analysis if non-adherence rates are between 65 and 85% (due to either non-adherence or withdrawal) [35]. This modelling focuses on estimating the effect of the interventions in the presence of non-adherence, but also incorporates adjustments for loss to follow-up associated with the intervention. A participant will be considered to be compliant with the intervention if they have adhered, that is, completed visits one, two and three with their physical activity coach.

As it is our intention to inform future confirmatory trials, we will explore the feasibility of outcomes as applied in this trial and this population. We will consider internal reliability of all summated scales and assess both construct and convergent validity of scales not previously used in HD. In particular, the ordinal scoring criteria for the PPT were developed for application in frail elderly people. The distribution of the timing measures will be specifically explored in order to either validate existing categories or define new categories specific for application in an HD population. Other measures that will be investigated for their application in HD include the IPAQ short form, SEIQol-DW, Life space assessment, ICECAP-A and the Lorig self-efficacy scale. Finally, we will compare the self-reported falls reported by patients at assessments (recall), and the falls reported by patients in their weekly health and falls diaries, in an attempt to validate falls reporting in this population.

A public sector, multi-agency perspective will be used for the economic evaluation. Exploratory health economics analyses will be conducted to investigate the patterns of participants’ health and social care service use, and the associated costs. Information on health and social care service use will be collected using a Client Service Receipt Inventory [23]. The cost of developing the intervention, training staff and the delivery of the intervention will be calculated. A cost-utility analysis using the EQ-5D-5 L [24] will be conducted to assess the incremental cost-effectiveness of the physical activity intervention compared to the social contact intervention, along with a cost-effectiveness analysis using the ICECAP-A [25]. We will further present a cost-consequence analysis of costs and a range of outcome measures for the participants. We will conduct two sensitivity analyses related to staff costs: firstly, we will test the effect of using staff at a higher and lower grade to conduct the training to deliver the intervention; secondly, we will test the effect of using staff at a higher and lower grade to deliver the intervention. We will also conduct a sensitivity analysis removing the cost of supplying a DVD player to participants.

### Process evaluation

At the end of the study, participants will be asked to complete an end of study questionnaire that focusses on their views of the trial and of the intervention. We will also attempt to contact any participants who drop out of the intervention to ascertain reasons for discontinuing. Coaching staff will be interviewed to gather their opinions on delivery of the intervention. Topics will include challenges to intervention delivery, perceived successes, barriers to implementation and suggestions on how to improve the intervention process. All the interviews will be digitally audio recorded and transcribed verbatim for further analyses. We will employ standard thematic analysis techniques, which is essentially a process of summarization, categorization and counting frequency of responses. The transcripts of interviews will be closely examined to identify themes and categories. Codes will be applied to these broad themes, which will then be broken down further into sub-codes. Agreement on concepts and coding will be sought between members of the research team to ensure reliability. We will identify commonly expressed themes as well as unusual cases. A proportion of the data (20%) will be coded by two different team members to check on reliability of the coding scheme. Interviewing will be iterative; where new themes emerge we will incorporate them into subsequent interviews.

### Ethical and regulatory considerations

Multicenter research ethical approval has been granted by South East Wales Research Ethics Committee B (approval number: 14/WA/0034). Site-specific approval has been granted by NHS Grampian (approval number: NRS14/NE120), Birmingham and Solihull Mental Health NHS Foundation Trust (approval number: NRR1272), North Staffordshire Combined NHS Healthcare Trust (approval number: CHC0101/RD), Sheffield Children’s NHS Foundation Trust (approval number: SCH/14/059), North Bristol NHS Trust (approval number: 3291), University Hospital Southampton NHS Foundation Trust (approval number: RHM NEU0229) and Central Manchester University Hospitals NHS Foundation Trust (approval number: R03666). Non-NHS site approval has been granted for Cardiff University (approval number: 14/WA/1151).

### Dissemination policy

Results of this trial will be reported in the first instance to the funders, and then communicated to participants and relevant health professionals in a series of open access publications within nine months of the end of the data collection. Authorship will follow the trial publication policy that has been developed based on British Medical Journal rules on authorship and contributorship.

## Discussion

While regular and sustained physical activity has the potential to benefit patients with neurodegenerative conditions such as HD, there are a number of disease-specific factors, which make it particularly challenging to establish regular exercise in this population. The intervention described in the current trial was developed following wide-ranging consultation with people with HD and their families, so as to give due consideration and to accommodate the known limiting factors in this population. The interpersonal interactions of the intervention are underpinned by the concepts of SDT placed within a broader BCW framework. The associated logic model aids in providing a consistent structure for intervention delivery and facilitates testing of short and potentially longer term outcomes in relation to the individual elements of the intervention. A major emphasis of the intervention is the development of highly individualized exercise plans. The exercise plans are therefore determined by individual needs and goals that may change with disease progression. Such an approach is important in a neurodegenerative disease so as to promote longer term disease management in a continually changing arena.

Any physical activity or exercise-based intervention relies heavily on the interaction between the provider (coach, therapist and/or trainer) and the participant. As a result, any improvement that may be seen may be, at least in part, attributed to social interactions. It is therefore important for intervention trials to incorporate active compactor arms to help reveal the specific effects of each intervention. Here, rather than having a traditional, usual care control group, the trial has a comparator arm which involves half of the participants receiving a series of social interaction sessions which are time-matched with the coaching sessions delivered in the intervention group.

We will consider the acceptability of the interventions to participants; the ability of centers to recruit participants; the willingness of participants to be randomized; the number of eligible participants; the acceptability of the measures and the time taken to collect data. In this trial, we have developed an innovative approach to data collection, monitoring and site support using iPads. In addition to using iPads as a data entry tool, site staff are able to use the device to hold regular video conference calls with research staff, enabling a greater level of day-to-day support and guidance. The iPads are also being used to make audio recordings of intervention sessions, in order to monitor fidelity. We have implemented a variety of measures to minimize loss to follow-up and missing data [36]. From a design perspective, we have explicitly considered the burden with respect to timings of assessments and intervention sessions. The intervention is delivered in the home, which may be another factor to minimize loss to follow-up. Adverse events are systematically documented and, if participants choose to discontinue the intervention, sites are encouraged to continue with outcome assessments wherever possible. Finally, data monitoring is extensive to ensure minimization of missing forms or data, and analysis will consider imputation methods as required.

Although measures of effectiveness and cost are being collected, this trial is not powered for full effectiveness analysis. The data gathered here will inform the design (including sample size calculations) and delivery of a confirmatory phase three trial. In order to ensure repeatability in confirmatory trials, reporting will follow the template for intervention description and replication (TIDieR) reporting guidelines for description of interventions [37], and the Consolidated Standards of Reporting Trials (CONSORT) extension for non-pharmacological interventions [38].

## Trial status

The trial opened to recruitment in May 2014, with recruitment expected to end in July 2015.

## Abbreviations

AE: Adverse event
ANCOVA: Analysis of covariance
BCW: Behavior Change Wheel
CACE: Complier-average Causal Effect
CONSORT: Consolidated Standards of Reporting Trials
CSRI: Client Receipt Inventory Service
EHDN: European Huntington’s Disease Network
HD: Huntington’s disease
ICECAP-A: ICEpop CAPability measure for Adults
IPAQ: International Physical Activity Questionnaire
ITT: Intention to Treat
NHS: National Health Service
NIHR CRN: National Institute for Health Research Clinical Research Network Portfolio
PAS: Perceived Autonomy Support
PI: Principal Investigator
PPT: Physical performance test
RAE: Related adverse event
SAE: Serious adverse event
SEIQoL-DW: Schedule for the Evaluation of Individual Quality of Life
UHDRS: Unified Huntington’s Disease Rating Scale.
